# Mapping the global influence of published research on industry and innovation

**DOI:** 10.1038/nbt.4049

**Published:** 2018-01-10

**Authors:** Osmat A Jefferson, Adam Jaffe, Doug Ashton, Ben Warren, Deniz Koellhofer, Uwe Dulleck, Aaron Ballagh, John Moe, Michael DiCuccio, Karl Ward, Geoff Bilder, Kevin Dolby, Richard A Jefferson

**Affiliations:** 1 Queensland University of Technology (QUT), Brisbane, Queensland Australia; 2 Motu Economic and Public Policy Research, Wellington, New Zealand; 3 Cambia, Canberra, Australia; 4 Research Services Division, ANU, Canberra, Australia; 5 CESifo Ludwig-Maximilians-Universität, Center for Economic Studies, Munich, Germany; 6 National Library of Medicine, National Institutes of Health, Bethesda, Maryland USA; 7 Crossref Oxfordw Centre for Innovation, New Road, Oxford, United Kingdom; 8 Medical Research Council, London, United Kingdom

**Keywords:** Business and industry, Scientific community, Intellectual-property rights, Research data

## Abstract

Measuring citations to scholarly works in the global patent literature enables assessment of the influence of published research on invention, industry and enterprise, at the individual and institutional level.

**Supplementary information:**

The online version of this article (doi:10.1038/nbt.4049) contains supplementary material, which is available to authorized users.

Public research is critical to the economy and to society. However, tangible economic and social impact occurs only when research outputs are combined, used and reused with other elements and capabilities, to deliver a product, practice or service. Assessing the context and influence of scholarship during the dynamic process of innovation rather than measuring *ex post* impact, may improve performance. With this aim, we have integrated and interconnected scholarly citations with global patent literature and created new tools to link the scholarly literature with the patent literature. The resulting tools we present here enable diverse stakeholders to freely evaluate the influence published research has on the generation and potential use of inventions as reflected by the patent system. We outline an evolving toolkit, Lens Influence Mapping, that allows assessment of individual scholarly works and aggregated outputs of authors for influence on industry and enterprise, as measured by citations within patents. This performance measure, applied at many levels and normalized by either research disciplines or technology fields of use, may expose and highlight institutional strength and practices, and guide future partnerships.

**Linking the scientific and patent literatures**

Public investment in science and technology is increasingly expected to demonstrate social and economic benefits^1,2,3,4^. Much effort has been focused on developing metrics, databases and methodologies for identifying and quantifying impacts of past investments and actions^3,4,5^. Understanding the connections between desired outcomes and research conducted many years earlier will at best provide signposts for current public policy or to help evaluate past policy. But in rapidly evolving and complex innovative environments, this *ex post* assessment provides limited guidance as to how to improve performance. We need tools that provide guidance throughout the trajectory of innovation that can increase the likelihood of impact in the future.

The term 'impact' implies causation. Research findings can strongly influence or enable the development of a product or service with economic value, but a particular piece of scholarship rarely 'causes' the delivery of such products or services. The concept of influence, rather than impact *per se*, reveals one-to-many relationships or many-to-many relationships, and surfaces opportunities to alter decisions and partnerships dynamically to enhance uptake of the scholarship.

For instance, contributions such as DNA and protein sequencing methods by Fred Sanger^6^, monoclonal antibodies by Kohler and Milstein^7^, or BLAST algorithms by Lipman, Altschul *et al*.^8^, have influenced and inspired tens of thousands of scholarly works and similar numbers of patented inventions that led to many products, without being themselves patented or monetized by the authors' institutions.

Few, if any, products in the marketplace are produced solely by public research institutions, and while spinouts may contribute some inventions, almost all products and services with social and economic impact require an innovation system^9^ and participation by diverse actors, to assemble complementary capabilities with diverse incentives and norms. Aligning these incentives, minimizing risk, decreasing transaction costs between these actors and motivating them in common pursuit of product development is thought to be the fundamental driver behind the evolution of companies, as articulated by Coase's “The Nature of the Firm”^10^. Optimum choices of persistent partnerships in product development will determine the effectiveness of any attempt to use science and technology as critical components in innovation. For outcome-oriented philanthropy or for public funding that seeks a deliverable product to improve the public good (e.g., a vaccine), these considerations should be paramount and should drive decisions. Regrettably, for much public investment, they are not paramount.

Modern innovation—the marketplace introduction of a new product or practice^11^—requires the aggregation of scientific and inventive inputs with other components, such as intellectual property (IP) rights, regulatory compliance, and manufacturing or marketing capabilities^12^, among many others. Can we learn from this evolution of company behavior to generalize interventions that make all innovation more effective and efficient, especially that for good public outcomes?

Here, we offer new open tools, including a new application, PatCite, for any party, not only to find current and past influences of scientific results on patent-based inventions, but also to map linkages that can guide their decision-making processes. The resulting interactive and dynamic maps show which scientific results, which scientists, and potentially, which institutions have influence over a subset of economic activity. We demonstrate how more granular knowledge through individual or aggregated scholarly works cited in patents can be used to discover and build novel linkages.

By analyzing resolved scholarly outputs from about 200 leading global research institutions over the past 35 years and their citations within the global patent corpus, we developed an international innovation and industry influence mapping (In4M) metric to measure and later rank institutional influence. The metric reflects a citation intensity measure of the patents citing third parties, weighted by the size of patent family over total resolved articles. Although the metric can be applied at individual, institutional, regional and country levels, and versioned as a standard or normalized measure to account for data variances, in this study, we applied it at the institutional level and normalized it based on ten categories of research disciplines and 35 technology fields of use, built systematically and based on the International Patent Classification (IPC) codes^13^. The metric, along with other supported tools, may reduce risks of wasteful investments and help align partners and incentives for social outcomes.

**Patents, prior art and scholarly citations**

The patent system provides a lens into the interfaces between knowledge and invention and invention and innovation. A patent can be granted for a new, non-obvious and useful invention, which has been adequately described to enable others to reproduce it. The invention must be placed in the context of relevant knowledge at the time of filing. The applicant and the examination process find and disclose documentary evidence of science and technology that preceded the work and are relevant or necessary to evaluate the work under review for patent^14^. This prior art can also comprise enabling literature, which lays the technical groundwork for the subsequent invention. Much of this disclosure takes the form of previous patents, but it also includes non-patent literature (NPL), such as scholarly publications.

Within the millions of documents (applications and grants) that constitute the patent corpus^15^ are many inventions of dubious or no value^16,17^, but in principle they signal patent holders' ambitions to either commercialize an innovation of which the patented invention is a part, or to stop others from commercializing such a product. Although the enforcement of patent rights is national in jurisdiction, research, production, manufacturing and trade are effectively global.

To protect an invention in multiple jurisdictions, it is generally necessary to file and prosecute in each location. It is expensive to file patents, and filing in many jurisdictions escalates costs considerably. Thus, such filings can be thought to indicate an expectation of economic return by the applicant based on this outlay.

Prior art disclosure requirements vary by jurisdiction. In the United States, a 'duty of candor' obliges applicants to submit any prior art known to them at the time of application filing^18^. In other major jurisdictions, such disclosure is optional and the onus of finding prior art is on the examination process, with examiners submitting the discovered literature to the data record^19^.

Nonetheless, prior art is recognized as potentially relevant, influential or enabling of the invention across many jurisdictions^20,21^, and efforts by the Task Force on Patent Statistics at the Organisation for Economic Co-operation and Development (Paris) have resulted in the formation of an international citation data set^19^ that includes patent and NPL citations. The curated data set is currently hosted by the European Patent Office (EPO; Munich), and exchanged with commercial providers as part of the DOCDB/XML project^22^.

Over 30 patent authorities voluntarily contribute citations to the EPO citation database. These can be in rich or poor structure format and their date coverage varies from one authority to another. The top four contributors are the United States Patent and Trademark Office (USPTO; Alexandria, VA, USA), World Intellectual Property Organization (WIPO; Geneva), EPO and the Chinese Patent Office (SIPO; Beijing)^23^. The provided data are usually aggregated, partially cleaned and normalized into field structures, among which are the NPL citation strings. Although some of these text strings may include scholarly literature, they are not resolved with unique identifiers and many of them are web URLs and in various non-scholarly forms, representing scholarly literature defined broadly to include, for example, conference proceedings and monographs.

The Lens began serving the scholarly citations^24^ in 2014, and by January 25, 2017, over 31 million NPL citations (resolved and unresolved with unique identifiers) were extracted from around 7.6 million patent records or 4.7 million simple patent families^25^ (Supplementary Table 1). A simple patent 'family' is a set of documents, often across multiple jurisdictions, that share a priority date and pertain to the same invention.

**Coverage by technology fields of use**

Using the technology classification groups or WIPO concordance table, which links IPC symbols with 35 fields of technology^26^, we split the 7.6 million patent records and examined the extent of coverage per technology sector by jurisdiction and over time. Results revealed a consistent pattern of relative frequency of inventions with citations across technology sectors and jurisdictions (Fig. 1).

**Figure 1:**
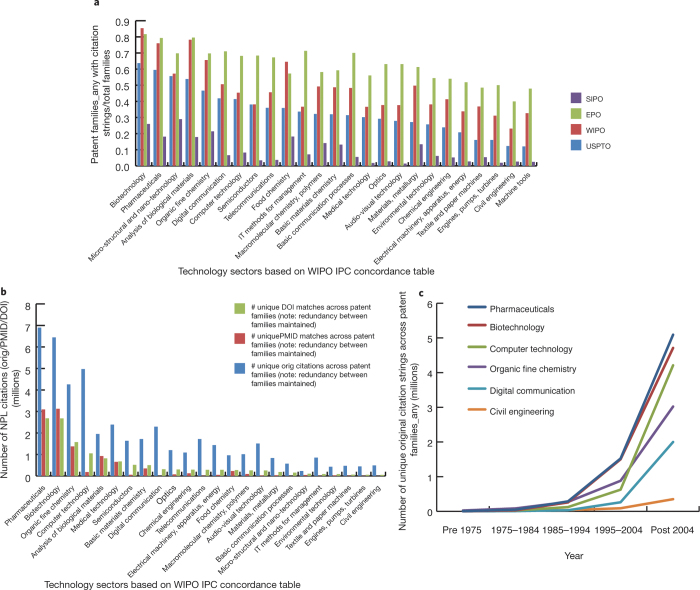
Frequency of inventions (simple patent families) with NPL citations. (**a**) Average of patent families with NPL citations per technology sector at the top four patent authorities: United States Patent and Trademark Office (USPTO), World Intellectual Property Organization (WIPO), European Patent Office (EPO), and Chinese Patent Office (SIPO). (**b**) Number of NPL citations per technology sector. (**c**) Unique NPL citations over ten-year intervals based on publication date in six illustrative IPC-defined technology sectors. Citation redundancy within simple patent families was removed.

An illustrative data set, limited by 25 selected technology sectors and extracted from the four main authorities, USPTO, WIPO, EPO and SIPO, shows that NPL citations are more common in technologies arising from research and in areas where industry has a heavy science-based R&D emphasis (Fig. 1a), particularly the life sciences industry (biotech) and pharmaceuticals, as reported previously, mainly on US patents^27,28^. However, the magnitude of the bias toward the life sciences varies somewhat across jurisdictions. Conceptually, the differences across sectors reflect some unknown combination of true differences in the influence of science on invention across fields, and in how prior art searches are conducted. The variation across jurisdictions suggests that variations in search practices are at least a portion of the story.

The observed skew in the total data set persisted in the resolved NPL data set, mainly in pharmaceuticals and biotech sectors (Fig. 1b) and was more visible over time (Fig. 1c). Even so, in fields with low relative numbers, such as civil engineering, the absolute number of NPL citations is not trivial (hundreds of thousands), so the potential exists even here to map knowledge linkages. As with variations across fields, the increase in NPL citations over time is likely a mixture of increases in the real influence of science on invention, and increases due to the greater diligence on the part of patent examiners. In the absence of an extrinsic measure of science influence, it is difficult to tease these apart. Hence, NPL metrics are likely to be more reliable indicators of influence at a point in time than in comparisons across time.

**Resolving NPL citations by identifiers**

In the Lens patent corpus, there are almost 54.87 million simple patent families (distinct inventions). Out of these, 4.7 million families (7.6 million patent documents) contain the 31.6 million NPL citations as strings of free text (Fig. 2). Without a standardized format requirement for the reporting of NPL citations in the global patent system, the usefulness of NPL data is constrained. To increase the value of this information, we challenged canonical, well-curated databases^29^, namely PubMed^30^ and Crossref metadata^31^, and resolved the poorly structured NPL strings into more uniform and standard formats through the use of open persistent identifiers (Supplementary Methods). During the analyses, we removed duplicate citations within a patent family; however, citations across patent families were counted more than once (Fig. 1b).

**Figure 2:**
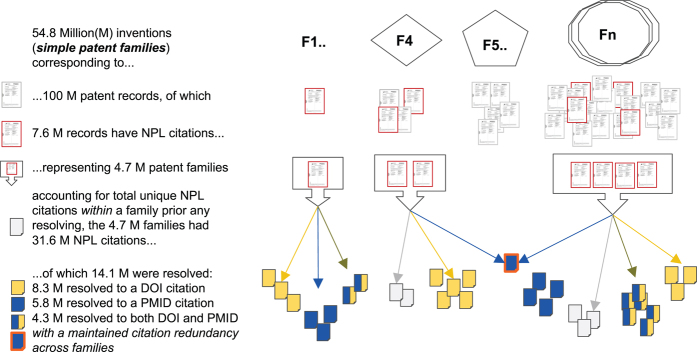
Summary of the overall resolving process of total NPL citations in the Lens through the use of open persistent identifiers. Resolving the 31.6 million NPL strings to either PubMed IDs or DOIs, or both, resulted in 14.1 million total resolved citations; 8.3 million resolved to a DOI citation, 5.8 million to a PMID, and 4.3 million to both IDs. Resolving and matching was done based on a matching score threshold of 0.9.

**Influence mapping tools in the Lens**

Cognizant that the role of scholarly citation in patents varies over jurisdictions, locations within the patent document and prosecution history^20^, the Lens offers users various entry points to define their search in terms of scientific and technological domain (patent classes), specific scholarly papers, specific authors or groups of authors (ORCID IDs).

Users can explore these documents in detail, to discover their commonalities, and the institutions or companies seeking or holding rights, and export their findings freely and within a secure and private space. In addition, we have enabled searching for NPL citations from those inventions with large family size (patent protection sought in multiple countries), which has been shown to be an indicator of likely economic significance^32^. This search/research resource allows a step to be taken toward understanding how various components in a particular innovation system are discovered and aligned, and by whom, where and when^33^ to determine influence. For instance, patents citing scholarly published works derived from the ORCID profile of Richard Jorgensen, a prominent molecular geneticist, can be viewed at https://www.lens.org/lens/search?q=citing_orcid_works%3A%28%220000-0002-0382-2371%22%29&predicate=%26%26&l=en.

To further connect scholarly work with inventions, the Lens provides PatCite^34^, a new tool that allows users to interrogate either resolved articles with unique identifiers or patent collections for analysis and sharing. A use case showing the influence mapping tool is provided in Box 1, Figure 3. PatCite enables influence mapping of a single article or a group of articles by means of a multi-stage citation-processing protocol that ensures quality matching and linkages between identifiers that point to a common article.

**Figure 3:**
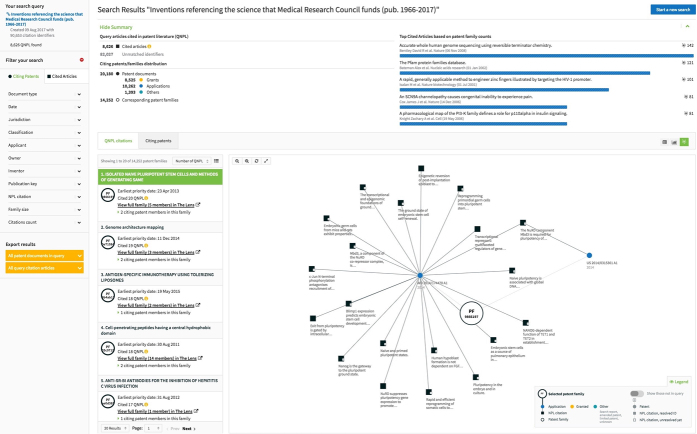
Search query results for inventions that referenced scholarship funded by the MRC from 1966 to 2017. The results can be explored further at https://www.lens.org/lens/patcite?uid=09873334-20ec-416d-82a4-3db1b37c0d63.

**Deriving an institutional influence metric and rank**

Using the resolved NPL citations, we have developed an influence metric that can be used to explore institutional and professional practices in translating scholarship to economic outcomes. To do so, we selected 200 global research institutions—degree and non-degree granting—based on whether they were named at least once as being among the top 100 research institutions by any of the following: the 2015 Nature Index, the Academic Ranking of World Universities (ARWU), Thomson Innovation or the 2015/16 Leiden ranking systems (with a few outlier institutions that did not rank among the top 100 in any of these ranking systems). For each institution, we required a comprehensive set of scholarly research outputs with persistent identifiers. As these are not apparently public open data, we sought and were granted limited permission by Clarivate Analytics to use their commercial data. The scope of this permission prohibits sharing the raw data openly, but allows the derived patent collections to be exposed on the Lens^35^.

In total, we had 11.8 million resolved scholarly outputs with unique identifiers extracted from the 1980 to 2015 time period (Supplementary Table 2) and 10%, on average, were matched in the Lens citation database. These were cited in 690,000 simple patent families (or 1.1 million patent records).

In considering the influence of an institution's scientific research on industry and enterprise, one may be interested in either an intensity or an aggregate measure. The intensity measure reflects an influence per unit of scholarly output, and the aggregate measure reflects both the intensity of influence and the volume of research generated, and thus attributes more influence to institutions with greater aggregate outputs, with a 'size bias' typical of other published rankings. As there is a need for more granular measures of influence that can be applied at many levels, and which can yield actionable results, we focus here on the intensity measure, the In4M metric.

We weight each unique citation by the size of the patent family, as this count is a proxy of a perceived economic value of the invention by patent applicants, as described, and also normalizes for variation in citation reporting between jurisdictions.

Applying the In4M metric per institution (patents citing third parties, weighted by family size, over total resolved articles), we found that smaller or more specialized institutions can sometimes outperform larger institutions (Supplementary Table 3). For example, The Scripps Research Institute (TSRI; La Jolla, CA, USA), a non-profit research institute, performs much better than more prominent institutions, such as Harvard University (Cambridge, MA, USA). TSRI has about 11 weighted patent citations per article, compared with 3 for Harvard.

The breadth of research disciplines differs across institutions and there is a recognized citation bias in life sciences technology sectors (Fig. 1). Differences among institutions could also reflect domain-specific patent drafting and examination practices, rather than true influence. To further investigate these initial results and understand the influence of one institution relative to that of others, we normalized the data set based on research disciplines.

**Normalization by research disciplines**

Using standard ISSN^37^ categorization, provided in the Crossref public application programming interface, we categorized all resolved articles in the data set according to their published journal category and then grouped these further into nine distinct research disciplines plus one group for all unassigned articles: Life Sciences, Chemistry & Materials, Physics & Electronics, Mechanical & Civil Engineering, Communications & IT, Social Sciences, Math, Earth Sciences, and Others (see Supplementary Table 4 for a detailed list of grouped categories). Within each discipline, total articles, third-party-citing patents weighted by family and the In4M metrics were determined.

On average, 70% of the articles were in the Life Sciences category, 17% in Chemistry & Materials, 14% in Physics & Electronics, and 5% or fewer in the other research disciplines (Fig. 4). Across the global data set, 12% were in the 'Others' category, with only 3% not assigned to any category.

**Figure 4:**
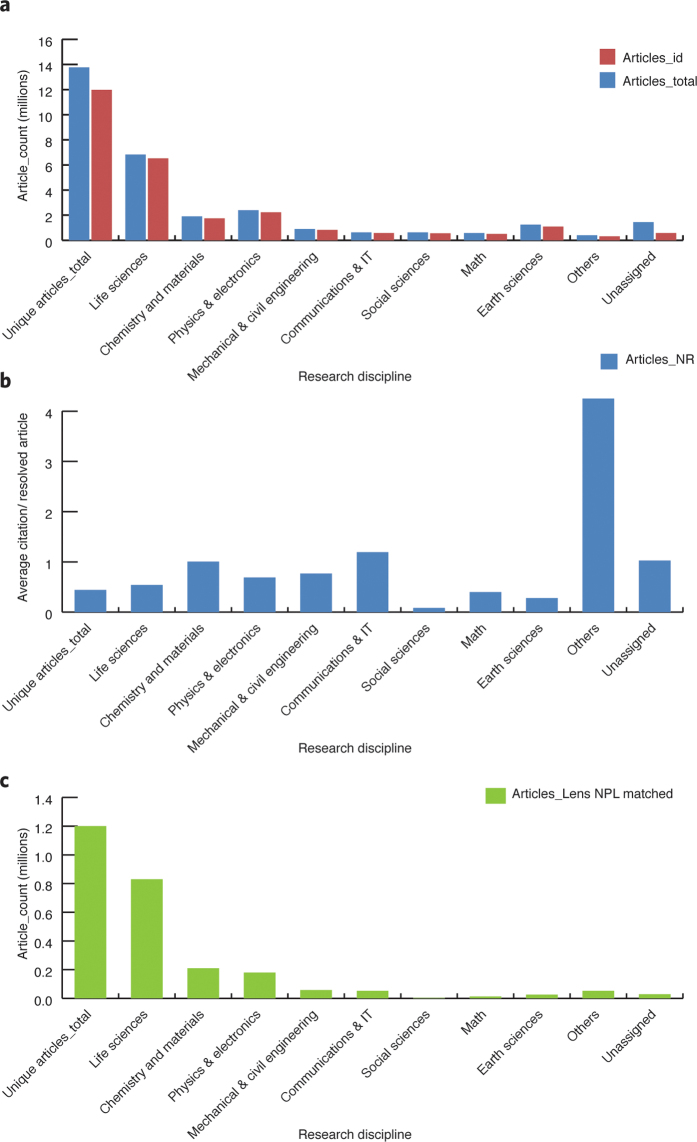
Normalized NPL by research discipline. (**a**) Distribution of total research institutions' articles (Articles_total), resolved articles with identifiers (Articles_id) based on nine research disciplines. (**b**) Average citation per resolved article based on non-redundant data sets and (**c**) Lens-matched non-patent literature (Articles_Lens NPL matched) per discipline. Each patent family member was counted only once per group, even if it cited multiple articles within a category. However, mixed categorization of articles covering multiple research disciplines was preserved.

To control for exogenous variations, we normalized the In4M metric for each discipline by the overall average citation per discipline across the non-redundant global data set (i.e., a resolved article or a citing patent document was counted only once). This process generated a relative In4M metric that we could use to compare institutions based on a specific research discipline.

Comparisons based on the relative In4M metric showed that variations across disciplines were relatively modest and differences were also modest, although some deviations were visible (Fig. 4b). Such results imply that a potential institutional In4M ranking system may be possible, based on normalization by research disciplines, as it would reflect a real economic influence, as perceived and acted upon by patent applicants, rather than being an artifact of patent practices.

**Ranking institutions based on a normalized In4M metric**

An institutional rank is mainly a reference to the overall influence of an institution on industry and enterprise, relative to other institutions. Such influence may reflect citation intensity based on either normalized scholarly research discipline (RD) or normalized technology fields of use (FOU) provided the potential use of the third-party-citing patents, weighted by family, are more revealing of the linkage between scholarship and patents.

To rank institutions based on normalized research discipline, we weighted the citation counts within a discipline by the overall citation average, summed the normalized aggregate citation counts across all disciplines and then divided by the total resolved articles. As expected, results showed institutional rankings favoring institutions, such as TSRI, and mainly those with strong life sciences programs (Table 2, column 3).

**Table 2:** Institutional rankings based on research discipline (RD) ^a^ or technology fields of use (FOU) according to WIPO concordance table

Research institution	In4M metric based on research disciplines (normalized citation counts/total resolved articles)	Normalized In4M rank_RDa	In4M metric based on FOU (normalized aggregate citation counts/total resolved articles)	Normalized In4M rank_FOU ^b^
Massachusetts Institute of Technology	9.48	3	39.82	1
The Scripps Research Institute	18.15	1	39.78	2
Rockefeller University	15.43	2	38.89	3
Carnegie Mellon University	4.75	71	31.66	4
Georgia Institute of Technology	4.86	64	27.10	5
Stanford University	7.02	9	26.39	6
Swiss Federal Institute of Technology Lausanne	5.14	41	26.26	7
California Institute of Technology	5.41	29	26.06	8
Rice University	5.62	22	23.70	9
Pohang University of Science and Technology	5.06	46	23.38	10
Weizmann Institute of Science	8.03	6	23.05	11
University of California, Berkeley	5.07	45	22.83	12
Lawrence Berkeley National Laboratory	4.76	68	22.41	13
Delft University of Technology	4.42	88	22.30	14
University of California, Santa Barbara	4.40	90	21.46	15
Korea Advanced Institute of Science and Technology	4.56	82	21.33	16
Hong Kong University of Science and Technology	3.81	118	21.01	17
Princeton University	4.74	72	20.92	18
Tokyo Institute of Technology	4.62	76	20.54	19
University of Massachusetts Medical School	8.70	4	20.09	20
University of Strasbourg	5.97	16	19.70	21
Purdue University	5.33	32	19.38	22
Pennsylvania State University	4.84	65	18.89	23
University of Massachusetts, Amherst	3.83	117	18.61	24
University of Texas Southwestern Medical Center	8.66	5	18.61	25
Technion Israel Institute of Technology	3.78	121	18.19	26
University of Southern California	5.56	24	18.19	27
University of California, San Diego	6.49	14	17.76	28
Gwangju Institute of Science and Technology	4.45	87	17.75	29
University of Illinois, Urbana-Champaign	4.17	100	17.57	30
Swiss Federal Institute of Technology Zurich	4.94	57	17.49	31
University of Massachusetts System	5.03	49	17.12	32
Tufts University	6.95	11	16.79	33
North Carolina State University	3.93	112	16.60	34
Washington University St. Louis	6.78	13	16.51	35
University of Washington	5.47	28	16.20	36
University of Utah	5.75	19	16.13	37
University of Cambridge	5.01	51	15.87	38
RIKEN	5.16	39	15.76	39
Dartmouth College	5.67	20	15.68	40
Cornell University	5.03	48	15.63	41
Northwestern University	5.08	44	15.63	42
University of Erlangen Nuremberg	4.55	83	15.62	43
Osaka University	5.34	31	15.53	44
Case Western Reserve University	5.80	18	15.52	45
Technical University of Denmark	4.28	98	15.30	46
University of California, San Francisco	7.04	8	15.15	47
University of Michigan	4.75	70	15.03	48
Baylor College of Medicine	6.84	12	14.92	49
University of Wisconsin	5.20	36	14.91	50

^a^This global ranking was released on QUT In4M site https://www.lens.org/lens/in4m#/rankings/global/locations on August 9, 2017. Data from the first two columns were featured in the *Nature Index* Supplement on August 10, 2017.

^b^Because of mixed categorization and the interdisciplinary nature of cited journals, ranking based on technology sectors seems to reveal more insight on particular scholarship strength in the institution relevant to industry.

To explore an alternative ranking based on potential uses of an invention, we classified each institution's third-party-citing patents, weighted by family, into the 35 technology fields of use (+1 for the unassigned group) classes as described above, and followed the same normalization process used for research disciplines. Comparing the two ranking systems, we observed position deviations among certain institutions (Table 2). Based on the normalized fields-of-use rank, the Massachusetts Institute of Technology (Cambridge, MA, USA) stepped to position one followed by TSRI and Rockefeller University for positions 2 and 3, respectively. As for Carnegie Mellon University (CMU; Pittsburgh) and Georgia Institute of Technology (Atlanta), their rankings shifted from 71 and 64 in the research-discipline-based ranking to 4, and 5 based on the fields-of-use ranking. As both institutions have strengths in non-life-sciences-based disciplines, these results suggest that ranking based on technology fields of use may highlight special institutional scholarship strengths as relevant to particular industry compared with that based on normalized research discipline.

Related to our finding, Ahmadpoor and Jones^37^ have recently shown that 80% of cited research is connected to future patents and 61% of patents link to prior scholarship, if linkage is defined on a network including indirect linkages. Although connectivity distances seem wider between industry and research institutions, the authors claimed that the relationship varies between the linear and nonlinear research models, depending on discipline and fields of use.

One can also envision an even more granular comparative ranking based on a specific FOU across institutions. Using the relative In4M metric per technology field, institutional strengths across all 35 technology fields of use can be compared with those for other institutions. Figure 5 shows an example comparing institutional research strengths of TSRI and CMU as relevant to inventions across the 35 technology fields. Although TSRI biggest influence appears linked to “Analysis of biological materials, Biotechnology and Pharmaceuticals” fields, CMU influence seems more tied to “IT methods for management, handling, Instruments-control, and Computer technology” fields.

**Figure 5:**
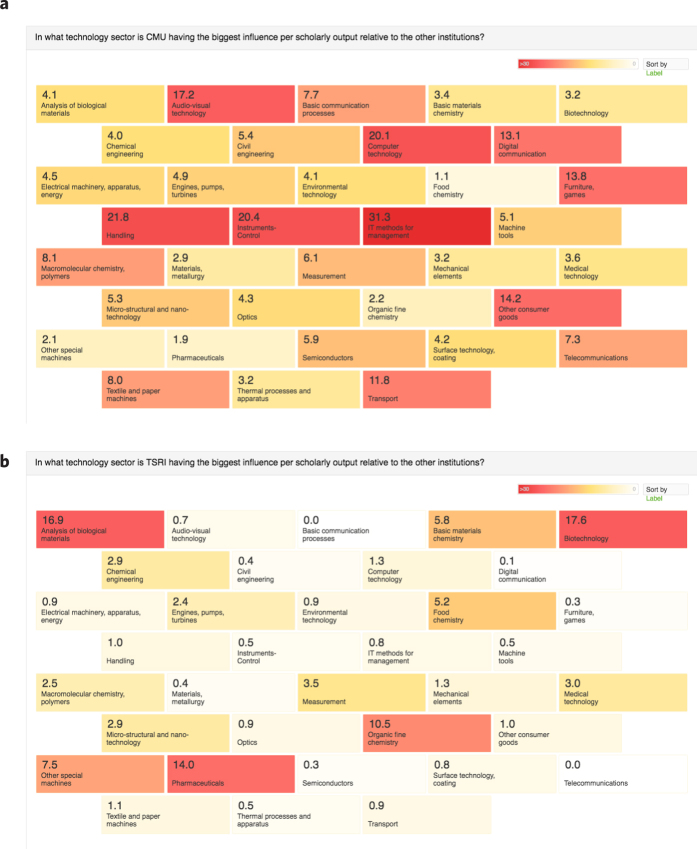
A heat map display of the strength of Influence (scale >30–0) in each of the 35 technology fields of use referencing scholarship from two institutions. (**a**) Data from the Scripps Research Institute. (**b**) Data from Carnegie Mellon University. Note the difference between the relative metric values in the biotech and IT methods for management fields between the two institutions. Such granularity can uncover linkages between scholarly works from a particular institution, regardless of its size, and a set of inventions that users can explore further.

**Discussion**

The route to economic and social impact from public research is complex, dynamic, risky and often unclear. Choosing the right partners and pathways is critically important, and requires mining metadata and knowledge from diverse corpora, including but not limited to science and technology scholarship and patents. Knowing which individuals and institutions are or could be actors in this journey, as well as what knowledge, capabilities and rights they may control, is essential. Similarly, surfacing and exposing potential incentives will provide the glue to hold such alliances in place.

Here, we describe a free and open platform and tools that can enable public and private parties to openly explore the relationships between scholarly works—including their authors (Box 2 and Table 1) and institutions—with innovative enterprise that participates in the global patent system. This is a step toward what we call 'Innovation Cartography', an open evidence-based mapping of the diverse capabilities, knowledge and institutions needed to create economic and social outcomes for any domain of innovation. This is currently done by larger private sector institutions as part of their business practice, but at great cost and with structural inefficiency of non-reusable data and insights. Our assertion is that much greater efficiency of the innovation system can be achieved by precompetitive sharing of knowledge where possible, and building upon open data.

**Table 1:** Influential authors highly cited in patents in three increasingly specific areas: nanobiotechnology; diagnosis by ultrasonic, sonic and infrasonic waves; and detection of arthritis

Technology ^a^	CPC code (patent families)	Number of patents (patent families)	Highly cited authors (patent citations)
Nanobiotechnology or nanomedicine	B82Y5/00	44,228 (6,199)	Robert Langer (285) Y. Wang (276) David M Goldenberg (207) Ralph Weissleder (206) J. Chen (197)
Diagnosis by ultrasonic, sonic or infrasonic waves	A61B8/00	8,726 (2,044)	Matthew O'Donnell (37) Gregg E. Trahey (35) David W. Roberts (23) Peter A. Payne (22)Q.X. Chen (21)
Detection of arthritis/rheumatoid arthritis	G01N2800/102	2,986 (479)	Walther J. van Venrooij (70) Ferdinand C. Breedveld (50) A. Robin Poole (49) Simon P. Robins (45) Josef S. Smolen (45)
CPC, cooperative patent classification.			

^a^The Lens Classification viewer allows users to filter their search by specific code and zoom in on any specific technology field to view most influential cited authors at one point in time.

For this to happen, we need to address and overcome current persistent challenges such as public accessibility to comprehensive scholarly data sets with full text and linkage to institutional information^38^, disambiguated authors and inventors' names linked to their publications, resolved additional NPL citations embedded within the body text of patents, and improved assignment and licensing data of issued patents. Similarly, continuing to build links to existing open initiatives that have compatible missions and expertise is important, including Crossref, ORCID, Open Corporates and many others.

Unlike other ranking systems, the institutional rankings described in this paper are built on an open and shareable In4M metric, and can evolve to accommodate improved data, and priorities. The metric can be explored at a detailed and granular level allowing public institutions and private partners to better discover each other, and to discover common ground (for more details, see Supplementary Note). Thus, informed, we are hopeful that more effective partnerships, built on transparent evidence and shared knowledge, can be forged, improving public benefit from science and technology.

*Editor's note: This article has been peer-reviewed*.

**Contributions**

R.A.J. designed the study and the In4M metric. O.A.J. designed and oversaw development of PatCite and QUT In4M apps. D.K., O.A.J., and A.J. performed all analyses. D.A., B.W., and D.K. built the Lens citation database and PatCite backend, D.K. provided data to build In4M site, J.M. and M.D.C. built Hydra for resolving patent citations against PubMed IDs, K.W. and G.B. provided Crossref support to resolve DOIs by the Crossref application programming interface. R.A.J., A.B., and U.D. contributed to the research institutions study. K.D. provided a PatCite user case scenario. Initial drafts were written by O.A.J. and R.A.J. and further edits were contributed by the other co-authors, mainly A.J.

Box 1: Assessment of impact of public funding by the UK Medical Research Council Evaluation of the impact or routes to impact of publicly funded research requires clarity in users (both commercial and noncommercial) and uses of emerging science and technology. In assessing the economic impact from such funding, the typical metrics of generation of IP and direct patenting of the UK's Medical Research Council (MRC)-funded scholarship can now be complemented by analysis of influence through publications being cited in the patent literature, particularly by third parties. In preliminary work, the MRC has used PatCite to explore this influence. Figure 3 depicts patent documents that reference published research funded by the MRC in the United Kingdom over the past ten years. There are >90,000 publications listed in PubMed that have been marked up as being linked to MRC funding. Of these papers, PatCite shows that 8,081 have been cited in the patent literature, linking to 19,767 different patent documents or 13,631 patent families. This immediately reveals a more significant MRC influence on the development of IP than was previously known, and allows the MRC to better understand which papers, researchers and thus which grants, are leading to or informing this type of innovation. It is also important to note that the vast majority (>96%) are papers published from 2007 onwards, with the systematic indexing of the funding starting in 2008. More pervasive influence may be discovered if one considers outputs from earlier MRC funding (e.g., that which gave rise to the seminal works of DNA sequencing from Fred Sanger's laboratory^6^ or monoclonal antibodies from the groups of Kohler and Milstein^7^).

Box 2: Most influential authors in life sciences technology fields To demonstrate the utility of NPL methodology to view most influential researchers in a particular life sciences technology field, we carried out searches by three subclasses of technologies using cooperative patent classification codes: nanobiotechnology (B82Y5/00); medical diagnostic devices using ultrasonic waves (A61B8/00), and methods to detect arthritis (G01N2800/102). Within each of the three selected patent collections, we examined the top-cited authors (Table 1).

## Supplementary information

Supplementary Text and Figures Supplementary Note, Supplementary Methods and Supplementary Tables 1 and 2 (PDF 134 kb)

Supplementary Table 3 Top 50 research institutions based on the normalized aggregate citation counts* (XLSX 98 kb)

Supplementary Table 4 List of grouped research fields. (XLSX 15 kb)
