# Supplementary material for: Mapping the global influence of published research on industry and innovation
Source: Nat Biotechnol. 2018 Jan 10;36(1):31–9. doi: 10.1038/nbt.4049 (PMC12094095; doi:10.1038/nbt.4049)
Supplement: Supplementary Text and Figures — Supplementary Note, Supplementary Methods and Supplementary Tables 1 and 2 (PDF 134 kb) [file 41587_2018_BFnbt4049_MOESM38_ESM.pdf]

Supplementary Note

Of the 100 million patent documents residing in The Lens, there are 7.6 million patent documents that contain non patent literature citations as strings of free text. These strings have been obtained from the DOCDB master documentation database.

As noted above, however, many of the patent documents pertain to the same underlying invention, both because within a jurisdiction there will be multiple documents for a given application (e.g. application, search reports, issued patent) and because the same invention can be the basis for applications in multiple jurisdictions. A patent 'family' is a set of documents, often across multiple jurisdictions, that all pertain to the same invention. As shown in Table X, the 100M patent documents represent almost 54.87 million families; of these 21.15 million are families in which at least one patent has been granted as of 1790 (ref 42). [1]

For 'metrics' purposes, it might be important to distinguish inventions with a granted patent from those without, but for our mapping purpose, we include all citations from all families, by the indicated technological activity by a given party in a given area, whether or not that activity has succeeded in a patent grant.

More than 4.7 million of the 55 million families contain at least one NPL citation. Multiple documents in the same family containing a citation to the same scholarly article do not meaningfully represent multiple citations. Hence, we eliminated duplicate citations within a family from our analysis. On this basis, there are 31.6 million total NPL citation strings, or about 6.7 strings per family (conditional on the family having at least one). **Supplementary Table 1** show more stats on the coverage of total NPL citation strings (resolved and unresolved with unique identifiers) in the Lens by January 25, 2017 and the resolved citations. Patent counts were based on simple patent families rather than individual document counts.

Supplementary Table 1

**Measure**	**Coverage in all patent**	**Coverage in patent families with a granted**

[1] https://www.lens.org/lens/search?s=pub_date&d=%2B&q=&dates=%2Bpub_date%3A18000101-20170228&types=Granted+Patent

Nature Biotechnology: doi:10.1038/nbt.4049

	families	patent
Resolved and unresolved NPL citation strings with unique identifiers		
Total number of patent families in The Lens	54,872,483	21,156,119
Number of patent families with a NPL citation string (whether resolved to an identifier or not)	4,701,336	3,425,91
Total number of NPL citations<br>US_NPL citations<br>WIPO_NPL citations<br>EPO_NPL citations<br>China_NPL citations	35,964,774<br>24,177,520<br>4,510,576<br>4,207,771<br>1,334,114	**Not determined**
Number of total NPL citations after duplicates within a patent family are eliminated	31,629,031	27,499,726
Average number of NPL citation strings per patent family (all)	6.7*	8.0
Maximum number of NPL citation strings in a patent family	3,238	3,238
Resolved NPL citation strings with unique identifiers		
Number of resolved citations to DOI*	8,302,553[†]	7,184,318
Average number of DOI citations per family	1.8*	2.1
Maximum number of DOI citations per family	608	608
Number of citations resolved to PMID*	5,864,716	4,900,595
Average number of PMID citations per family	1.3*	1.4
Maximum number of PMID citations in a	773	773

Nature Biotechnology: doi:10.1038/nbt.4049

family		

* A wide variation in number of citations per patent family was observed.

†Estimated overlap between DOI and PMID is 4.3 million

Supplementary Methods

PubMed is a highly enriched, standardized bibliographic dataset of the majority of the scholarly literature in the life and health sciences, each article of which is provided an unambiguous persistent identifier, called a PMID. Current holdings exceed 25 million records, and most include rich value-added data such as Medical Subject Headings (MeSH), textual data such as abstracts, links to primary articles, institutional affiliations etc. To support enhanced NPL citation resolution, NCBI developed a new indexing engine called Hydra. The goal of Hydra is to provide accurate resolution of query text to existing citations while minimizing the rate of false positive matches.[2] The focus of Hydra is on text available from abstracts, titles, author lists, and journal imprints (see next section below)

Crossref is a non-profit industry association that - in collaboration with its thousands of publisher members - enables the assignment and resolution of unique, persistent and unambiguous Digital Object Identifiers (DOIs) to scholarly work. Members of Crossref include both for-profit and non-profit, open and proprietary publishers, and their holdings currently exceed 80 million records. Assignment of DOIs extends into all research disciplines, including life sciences, physical, chemical, mathematical, economic and social sciences; it also includes books, monographs, conference proceedings and other works that are often important as prior art.[3] Resolving of NPL citations was done through the use of Crossref API.[4]

Description of the Hydra algorithm for Citation Matching

Hydra considers all abstracts from the publicly accessible medical and biological literature as provided through PubMed. From the text, all single words and two- and three-word phrases are extracted and included in the final dictionary. For each term, the initial field is

[2] Navarro, G. A guided tour to approximate string matching. *ACM Computing Surveys* **33**, 31-88, doi:10.1145/375360.375365 (2001).

[3] Paskin, N. Digital object identifiers for scientific data. *Data Science Journal* **4**, 1-9 (2005) accessible at http://www.doi.org/topics/050210CODATAarticleDSJ.pdf

[4] http://search.crossref.org/help/api - see /links endpoint

Nature Biotechnology: doi:10.1038/nbt.4049

remembered for purposes of ranking. Stop words are in general included; however, all two- or three-word phrases beginning with a stop word are dropped. We apply a limited term synonymy extraction, designed primarily to account for multiplicity among journal name representations. For example, “The New England Journal of Medicine” may be cited as “New Engl J Med”, or possibly “NEJM”. Author names are expanded to include variants of representation that include initials, full names, or last name only; dates are expanded to multiple possible forms, reflecting different representations in journal formatting. The full list of terms by documents are extracted into a series of postings files for indexed look-up.

There are two post-indexing stages used in the Hydra system to improve search performance in both execution time and fidelity. First, we extract multiple Bloom filters, used to exclude terms that are not present in the database. Second, we establish a set of per-field weights that are applied at run-time to rank results according to best-fit for citations. The per-field weights are determined using a tree-based boosting algorithm. We use a manually reviewed set of 5,000 user-provided queries as the basis for training, including both positive and negative citation searches from a set of user-supplied PubMed queries. Boosting requires the application of multiple independent algorithms to determine approximations of final field weights. Hydra currently employs two algorithms: a fuzzy-logic classifier employing a logistic regression and a ranking algorithm that uses field weights to establish relevance of articles. Field weights are initially biased toward English language abstracts and reviews from high-impact journals.

Searching within the Hydra system evaluates all incoming terms for presence within the postings file, using Bloom filters to exclude terms. All terms are permuted to induce a single-letter change (insertion, deletion, or change), generating a set of candidate terms with a Levenshtein edit distance of 1 from the initial set of terms. This expansion accounts for common artifacts and misspellings. Terms obtained through edit distance expansion are given a lower weight than initial terms. Search then proceeds using an estimator function that eliminates terms that are unlikely to affect the final ranking. Results are evaluated using a merge-sort-ranking algorithm that involves evaluating postings vectors in increasing order of size (shortest vectors first) and proceeds until at least three vectors or 10,000,000 documents are evaluated. As a result, high frequency single-word terms may not be evaluated at all, in preference to evaluating the two- and three-word phrases containing such high frequency words. Final scores are modified based on training weights, applied to fielded terms at merge-sort time.

Nature Biotechnology: doi:10.1038/nbt.4049

To support final citation matching, we further require that there is exactly one unambiguous match. The highest ranking item is then returned; in cases of score ties, all ties are returned, but the result would not be considered specific enough to qualify as a matched citation.

Additional readings

1. Friedman, J. H. "Greedy Function Approximation: A Gradient Boosting Machine". IMS 1999 Reitz Lecture. http://www-stat.stanford.edu/~jhf/ftp/trebst.pdf
2. Mason, L.; Baxter, J.; Bartlett, P. L.; Frean, Marcus (1999). "Boosting Algorithms as Gradient Descent". In S.A. Solla and T.K. Leen and K. Müller. Advances in Neural Information Processing Systems 12. MIT Press. pp. 512–518.
3. Bloom, Burton H. (1970), "Space/Time Trade-offs in Hash Coding with Allowable Errors", Communications of the ACM 13 (7): 422–426, doi:10.1145/362686.362692
4. Levenshtein, Vladimir I. (February 1966). "Binary codes capable of correcting deletions, insertions, and reversals". Soviet Physics Doklady 10 (8): 707–710
5. Navarro, Gonzalo (2001). "A guided tour to approximate string matching". ACM Computing Surveys 33 (1): 31–88. doi:10.1145/375360.375365.

Supplementary Table 2. Number of unique articles in the various datasets

**Dataset**	**Unique articles**
Articles_Clarivate Analytics_total	13,770,091
Resolved articles with identifiers	11,748,697
Resolved & cited articles in the patent literature	1,202,523
Average cited article /total resolved articles	0.10
Number of citing patents	1,117,712
Number of citing patents, weighted by family	4,659,652
Number of citing patent families	689,097

Nature Biotechnology: doi:10.1038/nbt.4049

Average citing patent/article (using resolved articles)	0.4

Supplementary Table 3. Top 50 research institutions based on the normalized aggregate citation counts*.[see Excel file]

*For the normalized aggregate citation measure, we first weighted citations in each of the 10 research disciplines and summed the normalized citations across all disciplines. To calculate the normalized In4M metric, we divided the normalized aggregate citation counts by the resolved articles. To view all 200 institutions' rankings, go to https://www.lens.org/lens/in4m#/rankings/global/locations

Nature Biotechnology: doi:10.1038/nbt.4049

Nature Biotechnology: doi:10.1038/nbt.4049
